# Supplementary figures and images for: Multi-Scale Gaussian Normalization for Solar Image Processing
Source: Sol Phys. 2014 Apr 8;289(8):2945–55. doi: 10.1007/s11207-014-0523-9 (PMC4938016; doi:10.1007/s11207-014-0523-9)

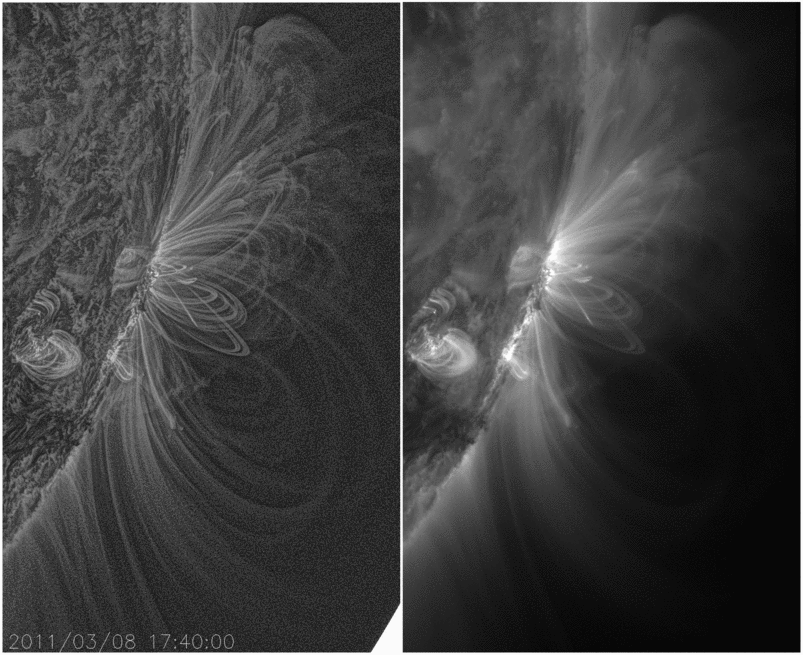

Supplement: Supplementary file 1 — (GIF 29.5 MB) [file 11207_2014_523_MOESM1_ESM.gif]

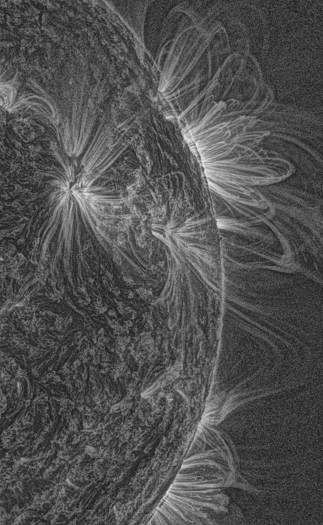

Supplement: Supplementary file 2 — (GIF 19.2 MB) [file 11207_2014_523_MOESM2_ESM.gif]

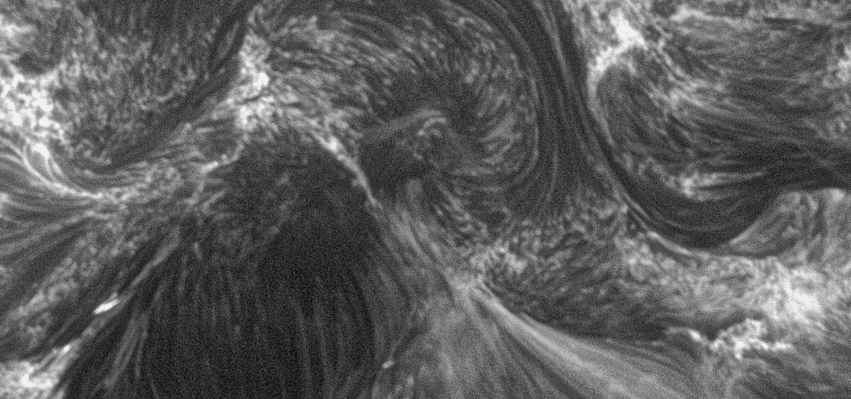

Supplement: Supplementary file 3 — (GIF 4.2 MB) [file 11207_2014_523_MOESM3_ESM.gif]
